# Predicting multigland disease in primary hyperparathyroidism using ultrasound and clinical features

**DOI:** 10.3389/fendo.2023.1088045

**Published:** 2023-03-27

**Authors:** Yanwen Luo, Siqi Jin, Yudi He, Song Fang, Ou Wang, Quan Liao, Jianchu Li, Yuxin Jiang, Qingli Zhu, He Liu

**Affiliations:** ^1^ Department of Ultrasound, Peking Union Medical College Hospital, Chinese Academy of Medical Science and Peking Union Medical College, Beijing, China; ^2^ Key Laboratory of Endocrinology, Department of Endocrinology, National Commission of Health, Peking Union Medical College Hospital, Chinese Academy of Medical Science, Beijing, China; ^3^ Department of General Surgery, Peking Union Medical College Hospital, Chinese Academy of Medical Science and Peking Union Medical College, Beijing, China

**Keywords:** multigland disease, nomogram, ultrasound, primary hyperparathyroidism, prediction

## Abstract

**Background:**

The identification of multigland disease (MGD) in primary hyperparathyroidism (PHPT) patients is essential for minimally invasive surgical decision-making.

**Objective:**

To develop a nomogram based on ultrasound (US) findings and clinical factors to predict MGD in PHPT patients.

**Materials and methods:**

Patients with PHPT who had surgery between March 2021 and January 2022 were consecutively enrolled to this study. Biochemical and clinicopathological data were recorded. US images were analyzed to extract US features for prediction. Logistic regression analyses were used to identify MGD risk factors. A nomogram was constructed based on these factors and its performance evaluated by area under the receiver operating characteristic curve (AUC), calibration curve, Hosmer-Lemeshow tests, and decision curve analysis (DCA).

**Results:**

A total of 102 PHPT patients were included; 82 (80.4%) had single-gland disease (SGD) and 20 (19.6%) had MGD. Using multivariate analyses, MGD was positively correlated with age (odds ratio (OR) = 1.033, 95% confidence interval (CI): 0.190–4.047), PTH levels (OR = 1.001, 95% CI: 1.000–1.002), multiple endocrine neoplasia type 1 (MEN1) (OR = 29.730, 95% CI: 3.089–836.785), US size (OR = 1.198, 95% CI: 0.647–2.088), and US texture (cystic-solid) (OR = 5.357, 95% CI: 0.499–62.912). MGD was negatively correlated with gender (OR = 0.985, 95% CI: 0.190–4.047), calcium levels (OR = 0.453, 95% CI: 0.070–2.448), and symptoms (yes) (OR = 0.935, 95% CI: 0.257–13.365). The nomogram showed good discrimination with an AUC = 0.77 (0.68–0.85) and good agreement in predicting MGD in PHPT patients. Also, 65 points was recommended as a cut-off value, with specificity = 0.94 and sensitivity = 0.50.

**Conclusion:**

US was useful in evaluating MGD. Combining US and clinical features in a nomogram showed good diagnostic performance for predicting MGD.

## 1 Introduction

Primary hyperparathyroidism (PHPT) is a common endocrine disorder, presenting as abnormal bone metabolism and hypercalcemia. Approximately 80% of patients with PHPT have a single parathyroid adenoma, while 15%–20% have multigland disease (MGD) (1). Parathyroidectomy is the only curative and definitive treatment for PHPT (2, 3). Surgical procedures for PHPT have evolved considerably in recent years, thanks to the development of preoperative localizing tools and intraoperative PTH(io-PTH). Minimally invasive and unilateral surgical approaches have replaced traditional bilateral neck exploration as they leave smaller scars, reduce the incidence of postoperative hypocalcemia, lower recurrent laryngeal injury rates, and shorten hospital stays, concomitant with high curative rates (4–7). Thus, accurate preoperative MGD localization is beneficial for minimally invasive surgical decision-making (5, 8).

For preoperative location, ultrasound (US) and technetium-99m sestamibi are the first lines of imaging (9). However, sensitivity for MGD is limited to 15%–35% for US and 30%–44% for technetium-99m sestamibi imaging (10). Underestimated MGD may lead to incomplete surgical excision and cause persistent and recurrent hyperparathyroidism (11). Previous studies have explored MGD risk factors and developed prediction models improving both single-gland disease (SGD) and MGD identification outcomes. CaPTHUS is a scoring system for SGD and combines preoperative biochemical and imaging results to generate a positive predictive value (PPV) of 100% for a total score ≥ 3 (12). The Wisconsin Index (WIN) predicts the probability of additional hyperfunctioning glands during surgery and uses preoperative parathyroid hormone (PTH) levels and lesion weight during surgery (13). Their clinical values have been validated (14, 15). However, neither of them can be used to improve the efficacy of MGD diagnosis preoperatively. Ultrasound (US) is a preferred option for the preoperative localization of PHPT as it displays high sensitivity, is noninvasive and radiation-free. To date, no studies have evaluated specific US features and performances when combined with clinical data to predict MGD. In this study, our aim was to identify US features associated with MGD and assess the predictive value of combined US and clinical data in determining an MGD in PHPT patients.

## 2 Materials and methods

### 2.1 Patients

This retrospective study was approved by the Institutional Review Board of Perking Union Medical College Hospital (K2448). Informed written consent was obtained from all participants.

All patients with PHPT who underwent parathyroidectomy at our hospital between March 2021 and January 2022 were enrolled. Inclusion criteria: 1) diagnosed as primary hyperparathyroidism (PHPT). PHPT is diagnosed after comprehensive evaluation of biochemical testing. Classic PHPT presents as elevated serum calcium levels and serum parathyroid hormone (PTH) levels (calcium > 2.5mmol/L; PTH > 65pg/mL) (2).; 2) successful surgery (normal calcium levels at least 6 months after surgery). Exclusion criteria: incomplete calcium and PTH levels, US examinations, or pathological results.

**Figure 1:**
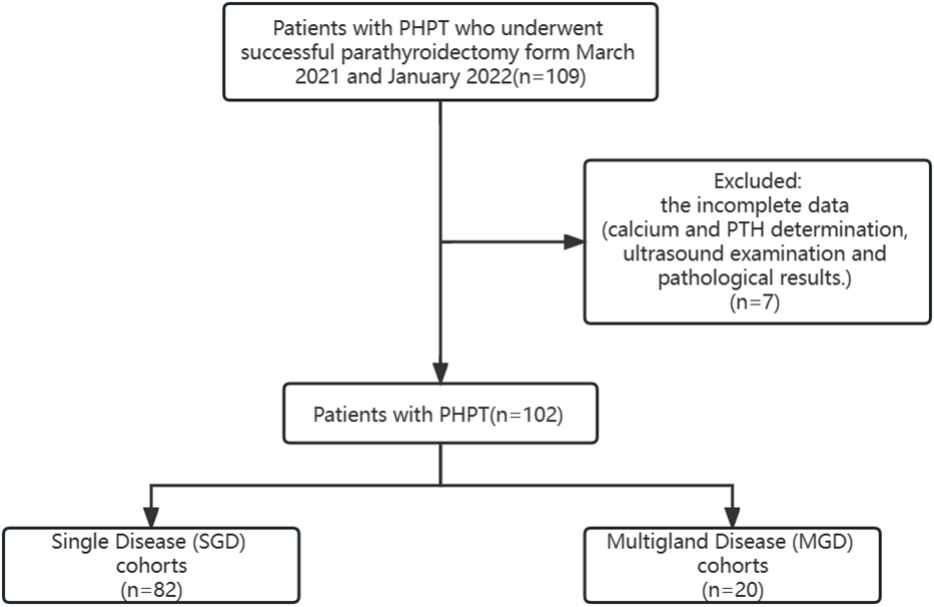
Study flowchart.

Based on operative records and pathological results, the cohort was divided in SGD group and MGD group. A study flowchart is provided (**Figure 1**).

Demographic and biochemical data were reviewed, including age, gender, symptoms, preoperative PTH and calcium levels, pathology reports, and operative notes. Of note, patients were defined as “symptomatic” if they presented with at least the one of the following symptoms: skeletal symptoms (bone pain and fractures), urinary symptom (nephrolithiasis), or gastrointestinal symptoms (anorexia, nausea, and constipation).

### 2.2 US image analyses

Before surgery, patients underwent US neck scanning by an experienced radiologist. US examinations were performed using IU22 (Philips Medical Systems, Royal Philips Electronics, The Netherlands) and Aixplorer (SuperSonic Imagine, Aix-en-Provence, France) with linear probes (3–12 MHz, centered at 10 MHz). For suspicious lesions, US grey scale images of both the longitudinal section and cross-section were acquired. The largest US size of the dominant candidate lesion in the longitudinal section was measured. The location, including the side and quadrant, was labeled. Patient imaging data were independently reviewed and selected for further analysis by two experienced radiologists, blinded to pathological data. US location and size were recorded. The US texture of suspicious lesions evaluated accordingly. If multiple lesions were identified by US, the dominant lesion was evaluated. US texture was mainly divided into two categories according to lesion echogenicity: solid (hypoechoic, isoechoic, and hyperechoic) and cystic-solid (lesions containing fluid (anechoic)) lesions.

### 2.3 Operative regimens

The surgical approach depended on preoperative localization findings; typically two concordant preoperative imaging studies were required. When first-line modalities, US, and parathyroid scintigraphy are negative or discordant, multiphase computed tomography (CT), positron emission tomography (PET) using 11C-methionine, and magnetic resonance imaging (MRI) are considered. Histopathological examinations were performed on all lesions removed during surgery and diagnoses made using World Health Organization classification criteria (16). Histopathological results were recorded as the gold standard.

### 2.4 Statistical analysis

Data were classified as categorical or quantitative variables. Categorical variables were reported as frequencies and percentages and analyzed using Pearson Chi-square tests. Quantitative variables were characterized by the median (minimum-maximum) or by the mean and standard deviation (SD) and compared using Student’s t-tests for parametric data and Mann-Whitney U or Kruskal-Wallis tests for non-parametric data where appropriate. Univariate and multivariate logistic regression analyses were performed on clinical and US characteristics. Based on multivariate analyses, risk factors were included in nomogram construction. Nomogram performance was evaluated by calculating the area under the ROC curve (AUC). Hosmer-Lemeshow tests and calibration plots were used to assess model fit. Decision curve analysis (DCA) was used to evaluate the clinical benefits of the nomogram.

A p < 0.05 value was considered statistically significant. Statistical analyses were performed using SPSS (SPSS 20.0, USA) and R 4.1.0 packages (https://www.r-project.org/), e.g., “rms,” “pROC,” “regplot”, and “ggDCA”. The inter-operator agreement of two readers was measured using κ values: κ < 0 (poor agreement); 0 < κ < 0.20 (slight agreement); 0.20 < κ < 0.40 (fair agreement); 0.40 < κ < 0.60 (moderate agreement); 0.60 < κ < 0.80 (substantial agreement); and 0.80 < κ < 1 (perfect agreement).

## 3 Results

### 3.1 Cohort clinical characteristics and US features

In total, data from 102 patients with PHPT who underwent parathyroidectomy were retrospectively collected. The mean age was 53 ± 12 years and 77.5% of patients were female. We observed that 52 patients had obvious clinical symptoms, including 30 with urinary, 26 with skeletal, and seven with gastrointestinal symptoms. The remaining 50 patients were asymptomatic. All patients underwent neck US, and 99 (97.1%) underwent 99mTc-MIBI in addition to US. Also, seven (6.9%) underwent 4D-CT, seven (6.9%) underwent PET-CT, and one (1.0%) underwent MRI. According to pathological findings, 82 (80.4%) patients had SGD and 20 (19.6%) had MGD.

Baseline clinical and US features of PHPT patients with SGD and MGD are shown (**Table 1**). Significant differences between SGD and MGD in terms of size (p = 0.006), US texture (p = 0.003), and pathology (p = 0.011) were recorded, but no significant differences were identified in terms of patient age (p = 0.08), gender (p = 0.335), symptoms (p = 0.992), and PTH (p = 0.113). The mean size of MGD patients was larger than that of SGD. Cystic-solid lesions were more often observed in MGD. In terms of pathological type, hyperplasia was more common in MGD. In the MGD cohort, 17 patients had double lesions and three had triple lesions. Of the 17 with double lesions, pathological findings included seven adenomas complicated with a hyperplastic gland (41.2%), four double adenomas (23.5%), three two-gland hyperplasias (17.6%), one double atypical adenoma (5.9%), one double carcinoma (5.9%), and one atypical adenoma complicated with an adenoma (5.9%). Three patients were confirmed with triple lesions, including three adenomas (n = 1), two gland hyperplasias with one adenoma (n = 1), and two atypical adenomas with one hyperplasia (n = 1).

**Table 1:** Demographics and clinical characteristics of patients with PHPT.

Characteristic		SGD	MGD	P-value
No. of patients		82 (80.39%)	20 (19.61%)	
Mean Age^†^		49.70 ± 13.02	55.30 ± 11.31	0.08
Gender				0.335
No. of women		62 (75.61%)	13 (65.00%)	
No. of men		20 (24.39%)	7 (35.00%)	
Symptoms				0.922
No		40 (48.8%)	10 (50.0%)	
Yes		42 (51.2%)	10 (50.0%)	
PTH*		152.00 (110.00-225.00)	194.85 (113.62-822.50)	0.113
Calcium^†^		2.76 ± 0.29	2.88 ± 0.58	0.206
US size^†^		1.82 ± 1.11	2.67 ± 1.60	0.006
Location				0.290
Right upper		16 (19.51%)	12 (28.57%)	
Right lower		28 (34.15%)	10 (23.81%)	
Left upper		18 (21.95%)	14 (33.33%)	
Left lower		19 (23.17%)	6 (14.29%)	
Ectopic		1 (1.22%)	0 (0.00%)	
US texture				0.003
Solid		80 (97.56%)	16 (80.00%)	
Cystic-solid		2 (2.44%)	4 (20.00%)	
Pathology				0.011
Adenoma		58 (70.73%)	19 (45.24%)	
Hyperplasia		15 (18.29%)	16 (38.10%)	
Atypical adenoma		9 (10.98%)	5 (11.90%)	
Carcinoma		0 (0.00%)	2 (4.76%)	

Except where indicated, the data refer to patient numbers. Numbers in parentheses are percentages.

PT, parathyroid hormone.

*Data are the median with interquartile ranges in parentheses.

^†^Data are the mean +/- standard deviation.

The inter-operator agreement for the ultrasonic feature (US texture) was 0.98.

### 3.2 Factors associated with MGD patients

Univariate and multivariate logistic regression analyses were performed to identify MGD risk factors (**Table 2**). Univariate logistic regression analysis showed that PTH levels (odds ratio [OR] = 1.001, 95% confidence interval (CI): 1.000–1.002, p = 0.007), MEN1 (OR = 20.250, 95% CI: 2.122–193.282, p = 0.009), US lesion size (OR = 1.556, 95% CI: 1.094–2.215, p = 0.014), and US texture (OR = 10.000, 95% CI: 1.686–59.314, p = 0.011) were MGD risk factors. Gender, age, preoperative calcium levels, and symptoms were not significantly different between groups (p > 0.05).

**Table 2:** Univariate and multivariate logistic regression analysis of factors associated with MGD.

Characteristic	Univariate analysis		Multivariate analysis	
	OR*	P-value	OR*	P-value
Gender				
female	1		1	
male	1.669 (0.585, 4.761)	0.3380	0.985 (0.190-4.047)	0.5148
Age	1.034 (0.992, 1.078)	0.1098	1.033 (0.985,1.092)	0.0828
PTH	1.001 (1.000, 1.002)	0.0072	1.001 (1.00,1.002)	0.0736
Calcium	2.140 (0.642, 7.135)	0.2155	0.453 (0.070,2.448)	0.0945
Symptoms				
No	1		1	
Yes	0.952 (0.358, 2.532)	0.9221	0.935 (0.257,3.365)	0.4034
MEN-1				
No	1		1	
Yes	20.250 (2.122, 193.282)	0.0090	29.730 (3.089-836.785)	0.9935
US size	1.556 (1.094, 2.215)	0.0140	1.198 (0.647,2.088)	0.2944
US texture				
Solid	1		1	
Cystic-solid	10.000 (1.686, 59.314)	0.0112	5.357 (0.499,62.912)	0.2232

*Numbers in parentheses indicate 95% confidence intervals.

OR, Odds ratio.

All risk factors clinically relevant to MGD were used in our model. The combined model (including clinical and US variables) showed that age (OR = 1.033, 95% CI: 0.985–1.092), MEN-1 (OR = 29.730, 95% CI: 3.089–836.785), US size (OR = 1.198, 95% CI: 0.647–2.088), and cystic-solid lesion (OR = 5.357, 95% CI: 0.499–62.912) were risk factors for MGD to some degree, and were used to develop a nomogram (**Table 2**).

### 3.3 Nomogram construction for MGD

**Figure 2:**
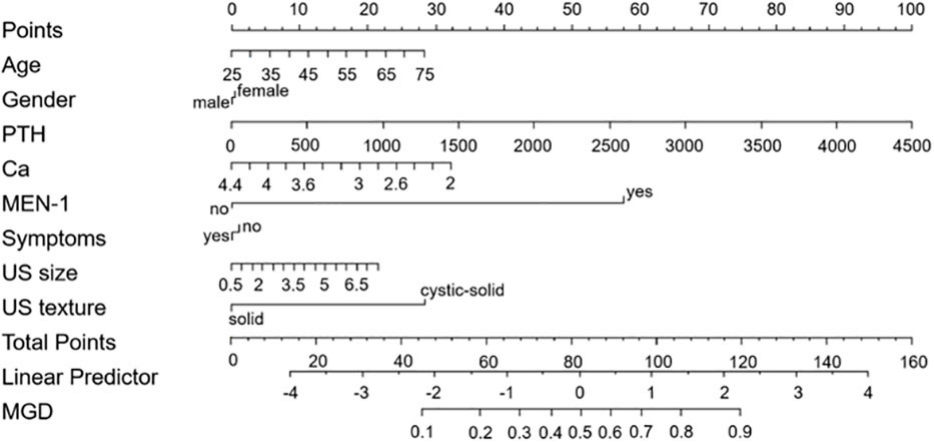
Nomogram predicting MGD probability in PHPT patients.

**Figure 3:**
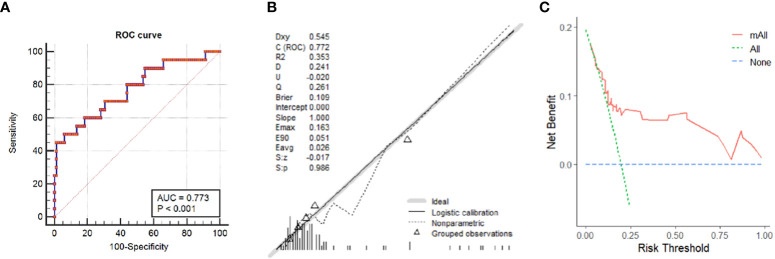
Nomogram prediction performance. **(A)** Receiver operating characteristic curve. **(B)** A calibration curve was generated by comparing observed and predicted probabilities in PHPT patients. **(C)** Decision curve analysis for MGD in PHPT patients.

Based on multivariate analysis results, a nomogram was built to visually predict MGD (**Figure 2**). The cut-off value was 65 points using the Youden’s index for MGD. For predicting MGD, the AUC was 0.773 (0.68–0.85) (**Figure 3A**). Calibration plots showed good consistency between observed and predicted probabilities (**Figure 3B**). Hosmer-Lemeshow tests showed adequate goodness of fit and yielded a chi-squares value of 6.68 (P = 0.67) for derivation cohorts. Furthermore, DCA (**Figure 3C**) showed a positive net benefit for patients with PHPT.

**Figure 4:**
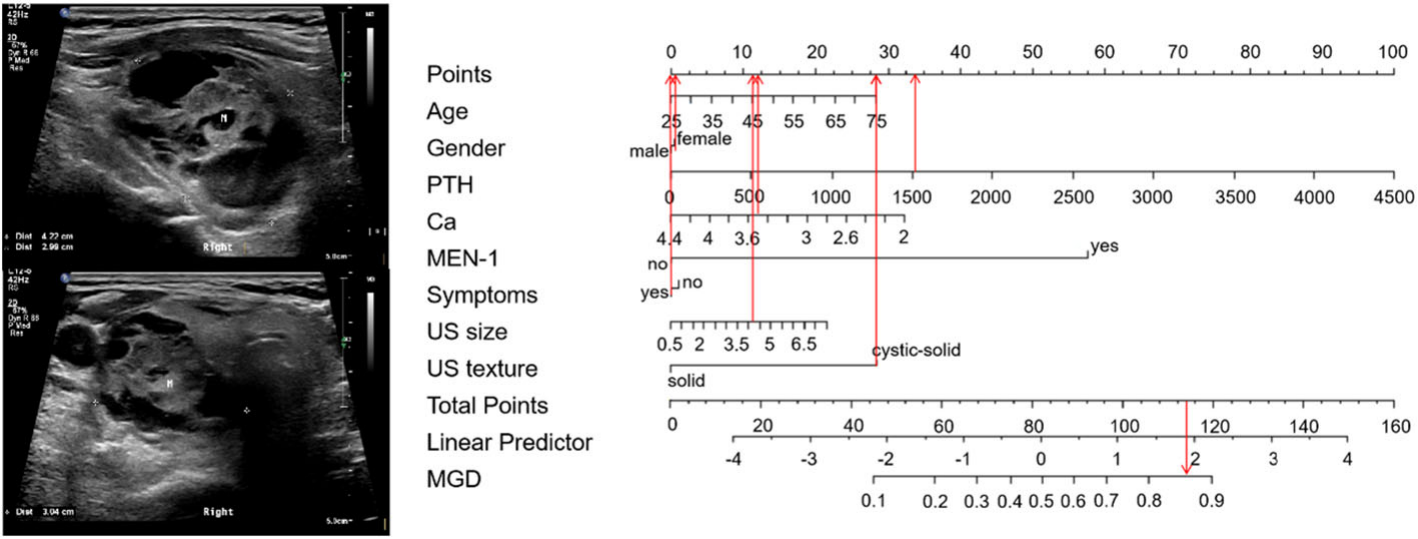
Using the nomogram for MGD - a clinical example. A 75-year-old woman with PHPT complained of bone pain in both lower extremities. PTH and serum calcium levels were 1526 pg/ml and 3.45 mmol/L, respectively. MEN-1 was not diagnosed. A neck ultrasound showed a 4.2 cm cystic-solid nodule in the posterior aspect of her right thyroid. The total score is 117, which corresponds to an MGD risk > 0.85. Pathology confirmed that there were two glandular involved (an adenoma with cystic degeneration on the right side, and a hyperplasia on the left side), with an adenoma and a hyperplasia.

**Figure 5:**
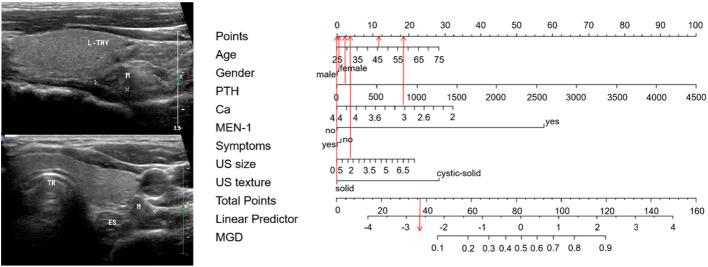
Using the nomogram for SGD - a clinical example. A 46-year-old man with PHPT complained of recurrent urinary stones. PTH and serum calcium levels were 201 pg/ml and 3.08 mmol/L, respectively. MEN-1 was not diagnosed. A neck ultrasound showed a 1.7 cm solid nodule in his left lower parathyroid area. The total score is 37, which corresponds to an MGD risk of < 0.10. Pathology confirmed an SGD (hyperplasia). L-THY, left thyroid; M, mass; TR, trachea; and ES, esophagus.

Sixty-five points was defined as the cut-off threshold, with specificity = 93.9% and sensitivity = 50%. Representative clinical examples showing nomogram-use for predicting MGD and SGD are respectively shown (**Figures 4**, **5**).

## 4 Discussion

MGD is an accepted diagnostic challenge in PHPT. Preoperative MGD localization in PHPT patients is key for minimally invasive surgical decision-making. In this study, we developed a nomogram based on clinical and US variables, which demonstrated good efficacy in discriminating MGD, with an AUC = 0.773 (0.68–0.85). In adopting 65 points as the diagnostic cut-off value, the nomogram showed good distinguishing capability, with specificity = 0.939 and sensitivity = 0.500. Good consistency was observed in the calibration curve, and the DCA curve showed efficient clinical applicability. Thus, our nomogram effectively predicted MGD and may be used to facilitate PHPT treatment strategies.

Previous studies established models for SGD and MGD predictions. CaPTHUS is a classical scoring system proposed by Kebebew et al. in 2006 (12) in which five standards are established to calculate SGD risk scores: (1) preoperative total serum calcium levels (≥ 3 mmol/L [≥ 12 mg/dL]); (2) intact PTH levels (≥ 2 times the upper limit of normal PTH levels); (3) sestamibi scan results positive for one enlarged parathyroid gland; (4) neck US results positive for one enlarged parathyroid gland; and (5) concordant sestamibi and neck US results (identifying one enlarged gland on the same side of the neck). Their results showed that with a score of ≥ 3, the PPV increased to 91% in predicting SGD. However, preoperative diagnostic efficacy for MGD has not been addressed. Mazeh et al. (13) defined the WIN, namely the multiplication of preoperative serum calcium by preoperative PTH levels, and found a strong correlation between the WIN and the gland weight. Therefore, a nomogram was built to identify the risk of additional hypersecreting parathyroid glands for each WIN category. MGD risk varied inversely with the weight of the first resected abnormal gland. During exploration for sporadic PHPT, a first abnormal gland <200 mg should heighten suspicion of MGD (17, 18). However, those methods could only be used intraoperatively to guide the decision. Recently, other imaging methods were used to identify hyperfunctioning glands; Sepahdari et al. (19) established a scoring model based on 4D-CT image dimensions, including the largest lesion size and number of suspicious lesions, and the combined scores with biochemical information, e.g., preoperative calcium and PTH levels and the WIN. As a result, 4D-CT MGD scores generated good specificity (81%–96%) and variable sensitivity (39%–64%) in predicting MGD. Sho et al. (20) prospectively validated 4D-CT scoring system efficacy and identified lower specificity (74%–88%) and similar sensitivity (32%–75%) when compared with retrospective data. Based on 4D-CT MGD scores, Bunch et al. (21) considered the size of the second-largest, high-confidence candidate lesion, either through estimated volume or maximum diameter values, to identify MGD by setting varying thresholds. However, the approach had relatively limited sensitivity for MGD (53%–67%). More important, the approach was only available for patients who showed a second lesion on 4D-CT.

In our combined model, not only the accompanied MEN-1 and cystic-solid lesion, but also the lesion size and patient age were associated with MGD. This observation indicated that associated MEN-1 represented MGD status, consistent with previous studies (22–24). In terms of the relationship between patient age and MGD, a previous study reported that older patients with PHPT were more likely to develop MGD (25). In our study, patient age was associated with MGD. Additionally, US size was also associated with MGD occurrence. Our results suggested that MGD patients tended to have larger lesions, with previous studies (16–18) reporting correlations between lesion size and MGD, however, these conclusion are controversial and require confirmation in further studies. Interestingly, in our study, US texture was also an important factor. Cystic-solid lesions are commonly found in MGD patients. This observation was not reported before and requires further investigation to explore the underlying pathology. Our nomogram incorporated refined US and clinical variables, and more accurately predicted parathyroid MGD in patients with PHPT when compared with situations when only clinical variables were considered. Importantly, the nomogram was relatively simple and easy to apply preoperatively.

Our study had some limitations. First, this was a retrospective and single-center study. The sample size was limited and nomogram efficacy requires comprehensive validation in larger sample and/or multicenter studies. Additionally, only gray-scale US image features were included, therefore semi-or quantitative US parameters, such as color Doppler, elastography, and contrast-enhanced US should be considered in future prospective trials.

## 5 Conclusions

In summary, our study indicated that certain US characteristics such as size and texture of the dominant lesion can be used to assess the potential risk of MGD. A novel nomogram was constructed by combing clinical and US characteristics to predict MGD in PHPT patients preoperatively. It could be helpful to assist treatment regimens for PHPT patients with MGD.

## Data availability statement

The raw data supporting the conclusions of this article will be made available by the authors, without undue reservation.

## Ethics statement

The studies involving human participants were reviewed and approved by Institutional Review Board of Perking Union Medical College Hospital. The patients/participants provided their written informed consent to participate in this study.

## Author contributions

HL, QZ, and YJ put up the research concept and design. SF, YH, and YL took part in the collection of the data. SJ, OW, and YL completed data analysis. QL and JL performed data interpretation. YL wrote the article. HL and QZ made revision of the article and provided the final approval of it. All authors contributed to the article and approved the submitted version.
